# Multidimensional Forced-Choice CAT With Dominance Items: An Empirical Comparison With Optimal Static Testing Under Different Desirability Matching

**DOI:** 10.1177/00131644221077637

**Published:** 2022-03-07

**Authors:** Yin Lin, Anna Brown, Paul Williams

**Affiliations:** 1 University of Kent, Canterbury, UK; 2 SHL, Thames Ditton, Surrey, UK; 3 Independent Researcher

**Keywords:** forced choice, computerized adaptive testing, multidimensional item response theory, Thurstonian IRT model, personality

## Abstract

Several forced-choice (FC) computerized adaptive tests (CATs) have emerged in the field of organizational psychology, all of them employing ideal-point items. However, despite most items developed historically follow dominance response models, research on FC CAT using dominance items is limited. Existing research is heavily dominated by simulations and lacking in empirical deployment. This empirical study trialed a FC CAT with dominance items described by the Thurstonian Item Response Theory model with research participants. This study investigated important practical issues such as the implications of adaptive item selection and social desirability balancing criteria on score distributions, measurement accuracy and participant perceptions. Moreover, nonadaptive but optimal tests of similar design were trialed alongside the CATs to provide a baseline for comparison, helping to quantify the return on investment when converting an otherwise-optimized static assessment into an adaptive one. Although the benefit of adaptive item selection in improving measurement precision was confirmed, results also indicated that at shorter test lengths CAT had no notable advantage compared with optimal static tests. Taking a holistic view incorporating both psychometric and operational considerations, implications for the design and deployment of FC assessments in research and practice are discussed.

## Forced Choice and Computerized Adaptive Testing

The forced-choice (FC) response format, where ranking responses are collected from simultaneous presentations of multiple items, is a frequently used response format in assessments of personality and other psychological traits. The popularity of the FC response format stemmed from its: (a) enhanced resistance against response biases and distortions when compared with a traditional “single-stimulus” (SS) rating scale response format (e.g., Cao & Drasgow, 2019; Cheung & Chan, 2002; Christiansen et al., 2005; Hirsh & Peterson, 2008; Jackson et al., 2000; P. Lee et al., 2019; Martin et al., 2002; O’Neill et al., 2017; Pavlov et al., 2019; Usami et al., 2016); (b) increased differentiations between the constructs being measured (e.g., Brown et al., 2017); and (c) good criterion-related validity (e.g., Salgado & Táuriz, 2014). An example FC question or “block” with two items is shown in Table 1. Each item in the block is an indicator for an underlying trait of interest. The format is further described as unidimensional FC (UFC) if items within the same block indicate the same trait, or multidimensional FC (MFC) if items within the same block indicate different traits.

**Table 1.** Example of a FC Block With Two Items.

Characteristic	Please choose the characteristic that is more like you
Quiet	√
Artistic	

For decades, assessments using the FC format faced issues with ipsative scores (Cornwell & Dunlap, 1994; Hicks, 1970; Johnson et al., 1988). An assessment’s scores are “ipsative” or “purely ipsative” if their total is a constant for all response sets, or “quasi-ipsative” or “partially ipsative” if the total score is not a constant but has some limited variance (Hicks, 1970; Meade, 2004). FC assessments often give rise to ipsative scores if classical test theory scoring is applied, because each FC block is given a fixed number of total points, corresponding to the available rank orders. Ipsativity leads to unnatural constraints in scale variance-covariance matrices (Clemans, 1966), thus distorting the scales’ factor structures and reliabilities (Meade, 2004), as well as compromising the scores’ interpersonal comparability (Johnson et al., 1988). Ipsativity is, therefore, a significant issue for measurement of individual differences. However, with the development of Item Response Theory (IRT) modeling of FC responses, scores from FC assessments are no longer ipsative (Brown, 2016; Brown & Maydeu-Olivares, 2011, 2013; Chernyshenko et al., 2009; Stark et al., 2005).

The development of FC IRT models not only made the extraction of information from comparative data more efficient (e.g., Brown & Bartram, 2009), but also opened up the possibility of computerized adaptive testing (CAT). CAT tailors an assessment to each and every individual in real time—the most informative questions for a candidate are presented, based on existing intelligence about them (e.g., their response to previous questions in the assessment, or their results from previous assessment occasions). CAT has demonstrated success in enhancing the measurement efficiency of FC assessments that utilize *ideal-point* items (Coombs, 1964). An ideal-point item is characterized by a curvilinear relationship between the probability of endorsement of the item and the underlying personality trait it indicates. In other words, there is a particular trait value (“ideal point”) at which the probability of agreeing with the item peaks, and deviations from this point in either direction lower the probability of endorsement. For example, “I am sometimes organized and sometimes forgetful” is an ideal-point item for Conscientiousness. A series of simulation studies of ideal-point FC assessments showed that adaptive assessments typically reach the same level of true score correlations at about half the test length of nonadaptive assessments (Joo et al., 2019; Stark & Chernyshenko, 2007, 2011; Stark et al., 2012). Several operational ideal-point FC CATs also emerged in the field of occupational psychology, including the Navy Computer Adaptive Personality Scales (Houston et al., 2006), the Global Personality Inventory—Adaptive (SHL, 2009–2014), the Tailored Adaptive Personality Assessment System (Drasgow et al., 2012), and the Adaptive Employee Personality Test (Boyce et al., 2014).

Despite the recent advancements in ideal-point FC CAT research, there is limited knowledge of the functioning of FC CAT with *dominance* items. A dominance item is characterized by a monotonic relationship between the probability of endorsement of the item and the underlying trait it indicates. In other words, as the trait value increases, the probability of agreeing with the item monotonically increases if the item is positively keyed, or monotonically decreases if the item is negatively keyed. For example, “I am organized” is a dominance item for Conscientiousness. Dominance and ideal point items exhibit different item characteristics, have different response processes, and demand different IRT models (Brown, 2015). It follows that the techniques for and the findings from ideal-point FC CATs cannot be immediately generalized to dominance FC CATs. As many existing content pools use dominance items, advancing research on dominance FC CAT will enable the utilization of validated historical content in the creation of new FC CATs, as opposed to needing to develop and validate new ideal-point items from scratch. Furthermore, dominance items present several practical advantages over ideal-point items. From a content development perspective, ideal-point items are harder to write and response to—attempts to write nonambiguous intermediate ideal-point items could lead to the introduction of response contaminants such as double-barreled conditional clauses, vaguely defined reference groups, or unintended contexts or multidimensionality (Brown & Maydeu-Olivares, 2010). From a content modeling perspective, ideal-point items are not invariant to reverse scoring (Maydeu-Olivares et al., 2006, p. 467), face greater challenges in item parameter estimation (Forero & Maydeu-Olivares, 2009), and are supported by fewer software options (Brown & Maydeu-Olivares, 2010). In contrast, the development and modeling of dominance items benefit from mature qualitative and quantitative best practice guidelines as well as data and software availability.

Although one recent study (Chen et al., 2019) did explore FC CAT with dominance items, it adopted the Rasch model that produces ipsative scores “with the constraint of zero sum across dimensions for every person” (W.-C. Wang et al., 2017), thus focusing on within-person profiling rather than cross-person comparison of assessment results. As for dominance FC CAT with normative (i.e., nonipsative) IRT scoring, either simulation or empirical research is scarce. To increase the understanding of dominance FC CAT, we conducted a simulation study of a multidimensional FC CAT using dominance items modeled by the Thurstonian IRT model (Brown & Maydeu-Olivares, 2011), and trialed this CAT with participants. This article presents our examination of dominance FC CAT in three aspects: (a) from a psychometric aspect, examining the measurement efficiency and utility of adaptive versus nonadaptive but optimal testing (via simulation and empirically); (b) from an applied psychology aspect, quantifying the impact of different social desirability balancing constraints on measurement (via simulation and empirically); and (c) from a psychological testing aspect, examining candidates’ perceptions and opinions about FC assessments (empirically). Arguably, the first aspect can be studied using simulations, as illustrated by many published studies on CAT. However, there is merit in studying the second aspect empirically, as simulations of socially desirable responding rely on many assumptions, which may not adequately represent the possible spectrum of actual candidate behaviors. Finally, the third aspect can only be explored through empirical engagement with participants.

This article is structured as follows. First, the psychometrics of dominance FC CAT using the Thurstonian IRT model is presented. Then, an empirical study is detailed, with results from a matching simulation study included alongside as theoretical benchmarks. The effects of adaptive testing and social desirability balancing on measurement precision, score distributions, and candidate perceptions are reported in separate subsections. Finally, implications for practice are discussed.

## CAT With the Thurstonian IRT Model

Several IRT models have been developed for the FC response format, for example, the probabilistic, multidimensional unfolding model (Zinnes & Griggs, 1974), the hyperbolic cosine unfolding model for pairwise preferences (Andrich, 1995), the multi-unidimensional pairwise preference model (Stark, 2002; Stark et al., 2005), and the Thurstonian IRT (TIRT) model (Brown & Maydeu-Olivares, 2011). Brown (2016) discussed the similarities and differences between such models and how they can be organized in a unified framework. For this study, the TIRT model is chosen. The TIRT model is able to handle multidimensionality, is flexible when modeling FC blocks of any size, and is compatible with the most commonly used dominance items. Moreover, the TIRT model has demonstrated great usability and utility in empirical applications, such as its ability to estimate item parameters from actual FC data (e.g., Brown & Bartram, 2009, 2009-2011; Brown et al., 2017).

The TIRT Item Response Function (Equation 1) describes the probability of preferring the first item in a pairwise comparison {i,k} (coded as $Y_{\left\{i,k\right\}}=1$ ), conditional on the respondent’s personality profile (represented by a latent trait column vector $\eta =(\eta_{1},\ldots ,\eta_{S}{)\;}^{T}$ with S dimensions) and the characteristics of the items being compared. The characteristics of item i (and likewise for item k ) are modeled through item parameters: $\mu_{i}$ is the mean utility of the item; $\lambda_{i}=({\lambda_{i}}_{1},\ldots ,{\lambda_{i}}_{S}{)\;}^{T}$ is a column vector of S factor loadings; and $\psi_{i}^{2}$ is the unique variance of the normally distributed residual error. As FC assessments tend to adopt factorially simple items that each indicates one and only one latent trait, the factor loading vector $\lambda_{i}$ usually contains one and only one nonzero entry ${\lambda_{i}}_{s_{i}}$ corresponding to the latent trait $\eta_{s_{i}}$ indicated by the item. For a full description of the model, including the modeling of FC blocks with more than two items, the interested reader is referred to Brown and Maydeu-Olivares (2011).

(1) $$p_{\left\{i,k\right\}}\left(\eta \right)\equiv P\left(Y_{\left\{i,k\right\}}=1|\eta \right)=\Phi \left(\frac{\mu_{i}-\mu_{k}+{\left(\lambda_{i}-\lambda_{k}\right)}^{T}\eta }{\sqrt{\psi_{i}^{2}+\psi_{k}^{2}}}\right)\equiv \Phi \left(z_{\left\{i,k\right\}}\right)$$

An IRT model serves two purposes in FC CAT. The first function of an IRT model is to enable the estimation of interpersonally comparable person trait scores from relative-to-self (or ipsative) responses resulting from the FC format. For this purpose, we chose the Maximum a Posteriori (MAP) estimator (Lord, 1986; Mislevy, 1986) with a multivariate normal prior reflecting the trait score distributions in the candidate population. The MAP estimates can be calculated by first analytically deducing the gradient of the log posterior function (see Appendix B in Lin (2020) for the full formula for TIRT), and then searching for trait values that set the gradient to zero. The Bayesian MAP estimator provides bounded and stable estimates even for short tests, making it particularly suited for use in early stages of CAT (Reckase, 2009).

The second function of an IRT model in FC CAT is to enable the parameterization of test items and traits to drive adaptive item selection. For this purpose, we chose the A-optimality item selector (Silvey, 1980), which minimizes the total error variance across all traits (i.e., minimizes the trace of the inverse Fisher Information Matrix). Past research has compared various multidimensional item selectors based on the Fisher Information Matrix and found A-optimality to offer good measurement efficiency (e.g., Lin, 2020; Mulder & van der Linden, 2009; Seo & Weiss, 2015). The TIRT Fisher Information Matrix for a pairwise comparison {i,k} can be deduced from Brown and Maydeu-Olivares (2017; Expression B.3) and takes the form of Equation 2.

(2) $$F_{\left\{i,k\right\}}\left(\eta \right)=\frac{{\left[\phi \left(z_{\left\{i,k\right\}}\right)\right]}^{2}{\left(\lambda_{i}-\lambda_{k}\right)}^{T}\left(\lambda_{i}-\lambda_{k}\right)}{p_{\left\{i,k\right\}}\left(1-p_{\left\{i,k\right\}}\right)\left(\psi_{i}^{2}+\psi_{k}^{2}\right)}$$

The test Fisher Information Matrix is then calculated by summing over the Fisher Information Matrices across all pairwise comparisons. Furthermore, the prior information of the covariances of the intended traits, as estimated during the test calibration, can be added to provide a total posterior Fisher Information Matrix (Brown & Maydeu-Olivares, 2017, equation B.9). The incorporation of prior information gives a Bayesian extension of A-optimality (Segall, 1996), which is especially helpful at the beginning of CAT where the test Fisher Information Matrix is singular.

## Method

### Item Bank

This study utilized an item bank for the HEXACO model of personality (Ashton et al., 2004; K. Lee & Ashton, 2008). The HEXACO model consists of six factors: Honesty-Humility (H), Emotionality (E), eXtraversion (X), Agreeableness (A), Conscientiousness (C), and Openness to Experience (O). A full description of the model is provided by K. Lee and Ashton (2009). The item bank (Lin, 2020) consists of 279 English adjectives, each measuring one and only one of the HEXACO factors. Each factor was indicated by between 24 and 81 adjectives. The items were pretrialed using a SS format and calibrated on a sample of 1,685 participants in the context of pre-employment assessment practice. The item parameters were calibrated in such a way that enabled subsequent use in a FC format with the TIRT model. This was achieved by aligning the arbitrary scaling of latent item utilities $t_{i}$ for all items to the six-point SS response categories (coded 1–6). Treating the observed item responses as continuous variables, a unidimensional CFA model was fitted to items for each of the six scales independently. For model identification, the scaling of the latent traits η were identified by fixing the trait means and standard deviations to 0 and 1, respectively. Such a simple unidimensional CFA model gives rise to three sets of parameters: factor loadings (directly mapping onto ${\lambda_{i}}_{s_{i}}$ in TIRT), intercepts (directly mapping onto $\mu_{i}$ in TIRT), and residual variances (directly mapping onto $\psi_{i}^{2}$ in TIRT). In order to compare with the difficulty/discrimination parameterization for typical IRT models, item discrimination parameters can be calculated as ${\lambda_{i}}_{s_{i}}\;/\psi_{i}$ . The absolute values of item discrimination parameters (i.e., $\left|{\lambda_{i}}_{s_{i}}/\psi_{i}\right|$ ) are summarized in Table 2. The item bank development process, as well as a full list of items and their associated IRT parameters, is provided by Lin (2020, Study 4, Table F4).

**Table 2.** Summary of the Absolute Values of Item Discrimination Parameters of the Adjectives Item Bank.

Scale	Mean	Minimum	Maximum
**H**onesty-Humility	0.49	0.15	0.78
**E**motionality	0.52	0.17	0.97
e**X**traversion	0.68	0.20	1.18
**A**greeableness	0.61	0.20	1.01
**C**onscientiousness	0.61	0.22	1.18
**O**penness to experience	0.49	0.20	1.03

### Empirical Study

#### Design and Procedure

A large sample (*N* = 1,440) was recruited online from a public-facing, pre-employment assessment practice website. Participants were invited to complete questionnaires to receive a personalized feedback report. After giving consent to partake in the research study, participants first completed a personality instrument consisting of 120 MFC pairs constructed from the HEXACO adjective item bank. Using a 2 × 2 between-subject design, participants were randomly routed into one of four design conditions: adaptive with lenient social desirability balancing (AL), adaptive with strict social desirability balancing (AS), nonadaptive but optimal with lenient social desirability balancing (NL), and nonadaptive but optimal with strict social desirability balancing (NS). One of the design factors, social desirability balancing, is considered important for minimizing response distortions in FC blocks (Krug, 1958). In lieu of social desirability estimates for the items, the items’ mean utility parameters (range 1.22–5.80, mean 3.61, *SD* 1.54) were used as proxies. The difference of item mean utility values within a pair was constrained to be no more than 0.5 in the strict conditions (AS and NS) or 1.0 in the lenient conditions (AL and NL). As for the other design factor, the adaptive conditions (AL and AS) always attempted to find the best MFC pair for the participants’ interim trait estimates (starting from the origin), leading to initially similar but subsequently divergent questions for different participants as their trait estimates evolved. The best MFC pair to present next was selected as follows (see Appendix A): (a) all possible MFC pairs of remaining items were created, (b) the MFC pairs not meeting the social desirability balancing constraint were removed, and (c) the remaining pairs were compared according to the A-optimality item selection criterion (with Bayesian extension) and the best one picked for presentation. The nonadaptive but optimal conditions (NL and NS), however, use static assessments that always target measurement at the origin. More specifically, the nonadaptive but optimal tests were created by applying the same steps as the adaptive algorithm, but fixing the interim trait estimates to the origin rather than re-estimating them (thus leading to static tests). As the latent traits were set to have zero means, the origin of the trait space represented a candidate in the target population that was average on every scale. In other words, the nonadaptive tests were optimized (following a local block-by-block optimization process but not necessarily globally optimal) for the average person in the target population. Participants were not informed of the random routing and did not know which route they were assigned to.

Following the FC instrument, each participant then responded to the HEXACO-PI-*R* (Ashton & Lee, 2009), a 60-item measure of the HEXACO model using traditional SS statements. The administration of the HEXACO-PI-R provided data to examine the construct validity of the FC measures, which are not reported here but is available from the first author upon request.

Following the FC and SS instruments, participants were presented with several follow-up questions asking about their experience with the two questionnaires. It was made clear to the participants that these questions were optional and would not affect their personality reports in any way, so that only the participants who were motivated to help with the research effort would complete them. The feedback questions reported here pertained to the FC questionnaire only (additional questions regarding comparison with the SS questionnaire are available from the first author upon request). They asked how frequently the participants noticed pairs of adjectives that were both like them or both unlike them (i.e., pairs with similar item utilities), to investigate whether adaptive item selection would lead to notably more difficult choices for the participants. The perception around social desirability of items was also investigated, through quantifying the perceived frequencies of FC adjective pairs with clearly unmatched social desirability. Finally, to gauge the perception of how fakable the FC response format was, participants were asked to imagine someone trying to answer the questions dishonestly to appear good, and rated how successful they thought that person would be in increasing their scores on the FC instrument.

Finally, participants were presented with six background questions. Gender, age, and self-rated English proficiency data were collected to capture the characteristics of the sample. English proficiency data also helped to ensure that the final sample consisted of participants who had good understandings of the English adjectives used in the FC measures. Then, to understand the mind-sets in which participants were completing the personality questionnaires, three questions explored whether their completion was a repeated participation, and whether their motivations to participate were associated with gaining experience for pre-employment assessments, finding out more about themselves, or something else.

The study website was built using JavaScript and integrated with custom R code. The website was hosted on an Amazon Web Services (AWS) server, which was specified to provide enough computational power for running simultaneous FC CAT sessions for multiple participants without causing notable delays in adaptive item presentation.

#### Data Cleaning and Final Sample

Due to the lack of participation control in online studies, extensive cleaning was applied to ensure data quality. Data cleaning removed (a) participants whose English proficiency level was below “Professional working proficiency”; (b) repeated completions by the same participants; (c) participants who had atypical motivations (i.e., other than “to practice for pre-employment assessments” or “to find out more about myself”); (d) participants who completed the study too quickly (<10 min, indicating lack of proper consideration) or too slowly (>2 hr, indicating presence of distraction during completion); and (e) participants with unusual or unreliable response patterns (e.g., when the majority of the rating scale was never used, when a particular response option was overused, when the responses had a very small standard deviation). The final cleaned sample (*N* = 1,150) was balanced in terms of gender (51.0% male, 44.8% female, 4.3% missing), and all working ages were represented (31.7% at 21–30, 32.0% at 31–40, 20.0% at 41–50, 8.7% at 51–60). About two fifths (39.1%) of the sample indicated that they had “native or bilingual proficiency” in the English language, a further third (32.0%) had “full professional proficiency,” whereas the remaining (28.9%) had “professional working proficiency.” Most participants (57.8%) spent between 20 and 40 min completing the study. All participants joined the study to practice for pre-employment assessments (87.4%) and/or to find out more about themselves (70.6%). With the random routing of different FC measures, each of the four conditions was completed by between 279 and 301 participants. All adaptive sessions reached the full test length of 120 FC pairs (i.e., there were no early test terminations caused by the lack of viable MFC pairs in the item bank).

### Simulation Study

A simulation study with settings mirroring the empirical setup was conducted to provide theoretical benchmarks for the empirical results. The simulations originally covered all four conditions (i.e., AL, AS, NL, and NS) of the empirical study. Moreover, following suggestions by an anonymous reviewer, two additional conditions that incorporated no social desirability constraint at all (i.e., adaptive with no social desirability balancing, nonadaptive but optimal with no social desirability balancing) were also simulated. Each condition was simulated on a sample of 2,000 simulees with a multivariate normal true score distribution (with covariances estimated during item development and calibration).

## Analysis Strategy

Analysis examined the effect of two design factors on three types of outcomes. The design factors considered were (a) adaptive versus nonadaptive but optimal item selection and (b) strict versus lenient social desirability balancing. The outcomes explored included (a) measurement precision, (b) score distributions, and (c) participant perception. Although a small number of predictions were made, most of the analysis was exploratory.

### Measurement Precision

To quantify measurement precision, standard errors of measurement (SEMs) were computed for each trait as the reciprocal of the square root of the posterior test information in direction of that trait (Brown & Maydeu-Olivares, 2011, Equation 25). In practical applications and interpretations of assessment scores, only the directions along the intended traits are of interest, which calls for directional information as the target measure. In addition to the SEMs, in the case of the simulation study where true scores were known, the correlation between true and estimated scores (CORs), as well as the root mean square errors (RMSEs) of the estimated scores, were also computed.

Adaptive measures were expected to achieve greater measurement precision, resulting in lower SEMs, higher CORs, and lower RMSEs. Lenient social desirability balancing placed less restrictions on FC block assembly, leading to more freedom in the tailoring of questions to individuals and thus better measurement in a pure theoretical sense (i.e., if the responses were affected only by latent trait values), expected to result in lower SEMs, higher CORs and lower RMSEs in the simulation study. However, it remained unclear whether this would be the case in the empirical study where socially desirable responding behaviors may be present. As highlighted by an anonymous reviewer, the effects of desirability constraints in simulation studies require a psychometric interpretation, whereas the effects of desirability constraints observed in empirical results require psychometric as well as psychological interpretations.

### Score Distributions

In the context of pre-employment assessments, certain score ranges are generally considered more favorable: high Honesty-Humility, low Emotionality, high Agreeableness, high Conscientiousness, and high Openness to Experience. The FC response format is designed to prevent socially desirable responding, with the strict balancing criteria expected to be more successful in doing so than the lenient balancing criteria. Therefore, conditions AS and LS were expected to have less favorable sample mean scores than conditions AL and NL. The adaptability condition, however, was not expected to affect mean scores.

### Participant Perceptions

Response frequencies for the feedback questions were summarized and compared across design conditions. It was anticipated that adaptive item selection/ strict social desirability balancing would result in more difficult choices, increasing the perceived frequencies of seeing adjective pairs that were equally like the participants/equally socially desirable, as well as lowering the expected success in faking good.

## Results

### Empirical Study

#### Measurement Precision

**Figure 1.**
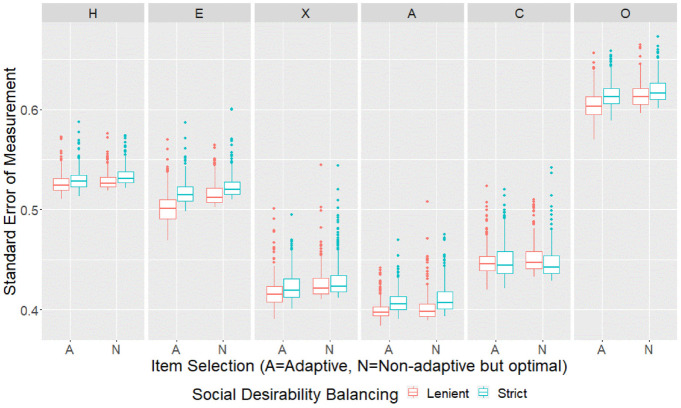
SEMs by Design Conditions for the Empirical Sample. *Note*. SEMs = standard errors of measurement.

**Figure 2.**
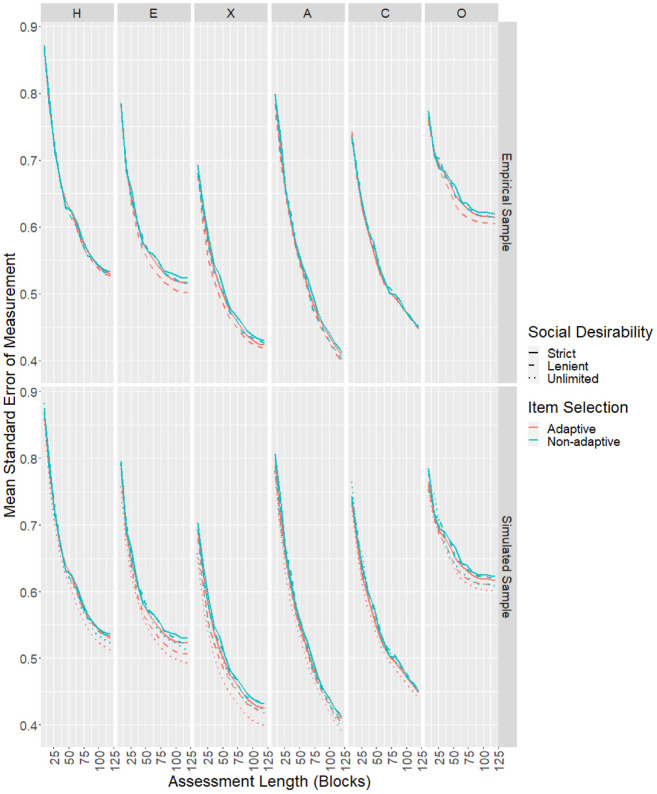
Sample Mean SEMs by Test Length and Design Conditions for the Empirical and Simulated Samples. *Note*. SEMs = standard errors of measurement.

**Figure 3.**
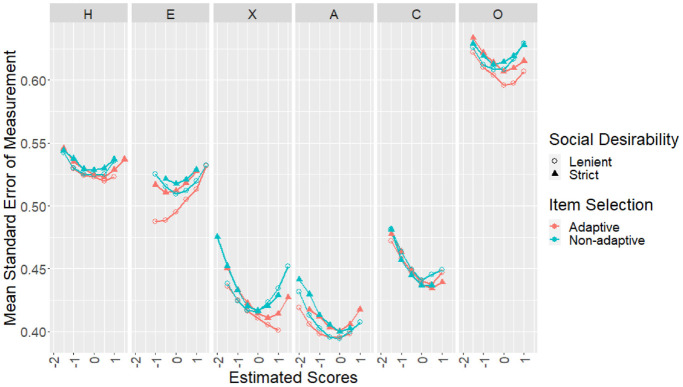
Sample Mean SEMs by Trait Values and Design Conditions for the Empirical Sample. *Note*. SEMs = standard errors of measurement.

**Figure 4.**
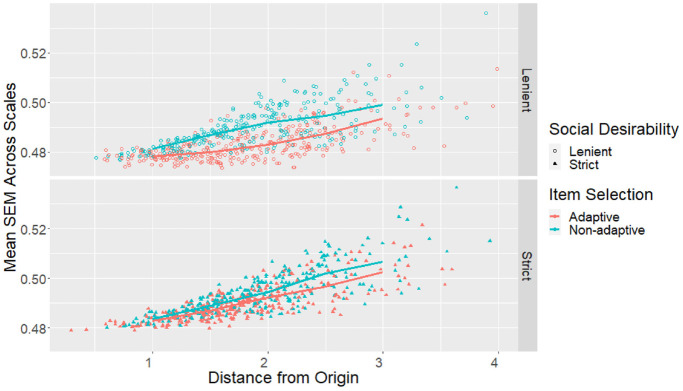
Profile Mean SEMs by Distance From the Origin and Design Conditions for the Empirical Sample. *Note*. SEMs = standard errors of measurement.

To model the effect of adaptive item selection and social desirability balancing on SEMs, a two-way analysis of variance (ANOVA) with type III sums of squares and unbalanced design was conducted (using the “Anova” function in the “car” package in R, Fox & Weisberg, 2019). Furthermore, the Tukey HSD test (using the “HSD.test” function in the “agricolae” package in R, de Mendiburu, 2020) was conducted to compare the mean SEMs across the four design conditions and place them into groups that are not significantly different. Analysis was conducted for each of the six scales independently using all cases in the sample. Results for all scales are summarized in Table 3 (ANOVA) and Table 4 (mean SEMs, with subscripts showing Tukey HSD adjusted significance group assignments). Visually, the full distributions of SEMs across all individuals in each of the four conditions are shown in Figure 1. The sample mean SEMs for each scale as the test progressed are shown in the top panel of Figure 2. The final achieved mean SEMs by estimated trait values are shown in Figure 3 (for each scale, participants were placed into bins of width 0.5 of the latent trait metric according to their estimated scores, and mean SEMs were plotted for bins with at least 10 participants). The individuals’ mean SEMs (i.e., average SEM across all six scales for each participant) were plotted against the Euclidean distance between their estimated score profile and the origin (i.e., the starting location of adaptive item selection) in Figure 4.

**Table 3.** Two-Way ANOVA of SEMs by Item Selection and Social Desirability Balancing.

Scale	Item selection			Social desirability balancing			Interaction		
	*F* (1, 1,146)	*p*	Partial eta^2^	*F* (1, 1,146)	*p*	Partial eta^2^	*F* (1, 1,146)	*p*	Partial eta^2^
**H**	8.02	.005	0.02	15.81	<.001	0.04	1.98	.160	<0.01
**E**	163.89	<.001	0.14	198.19	<.001	0.16	21.53	< .001	0.02
**X**	42.65	< .001	0.06	20.34	<.001	0.03	0.74	.391	<0.01
**A**	6.45	.011	0.02	59.78	<.001	0.10	0.37	.543	<0.01
**C**	5.85	.016	<0.01	0.36	.550	<0.01	7.49	.006	<0.01
**O**	86.56	< .001	0.08	74.89	< .001	0.07	8.73	.003	<0.01

*Note.* H = Honesty-Humility; E = Emotionality; X = eXtraversion; A = Agreeableness; C = Conscientiousness; O = Openness to Experience; SEM = standard errors of measurement. Partial eta squared effect sizes for type III ANOVA were computed using the “eta_squared” function in package “effectsize” (Ben-Shachar et al., 2020), with contrast coding handled by function “aov_car” in package “afex” (Singmann et al., 2021).

**Table 4.** Sample Mean SEMs by Design Conditions.

Scale	AL (*N* = 301)	AS (*N* = 288)	NL (*N* = 279)	NS (*N* = 282)
**H**onesty-Humility	0.527_c_	0.530_b_	0.529_b_	0.534_a_
**E**motionality	0.502_c_	0.517_b_	0.516_b_	0.524_a_
e**X**traversion	0.417_c_	0.423_b_	0.426_b_	0.431_a_
**A**greeableness	0.400_c_	0.408_b_	0.402_c_	0.411_a_
**C**onscientiousness	0.449_ab_	0.449_ab_	0.452_a_	0.447_b_
**O**penness to experience	0.605_c_	0.614_b_	0.615_b_	0.619_a_

*Note*. AL = adaptive with lenient social desirability balancing, AS = adaptive with strict social desirability balancing, NL = nonadaptive but optimal with lenient social desirability balancing, NS = nonadaptive but optimal with strict social desirability balancing; SEM = standard errors of measurement. For each of the six scales, mean SEMs with the same subscript letter are not significantly different.

Adaptive conditions tended to achieve significantly (Table 3) but only very slightly lower (Table 4) mean SEMs compared with nonadaptive but optimal conditions with the same social desirability balancing criteria. Only Emotionality, eXtraversion and Openness to Experience scales showed visible improvements when adaptive item selection was used (Figure 2). It appeared that the advantage of adaptive item selection was more prominent at certain trait values in certain scales (Figure 3). Regardless of design conditions, the score profiles further away from the origin tended to have larger mean SEMs compared with profiles nearer to the origin, but adaptive item selection helped to counter this effect (Figure 4).

Lenient social desirability balancing tended to achieve significantly (Table 3) but only slightly lower (Table 4) mean SEMs compared to strict social desirability balancing with the same item selection method. Lenient social desirability balancing was sometimes required for the advantage of adaptive item selection to emerge, and helped such advantage to appear earlier in the assessment process (Figure 2). With lenient social desirability balancing, the difference between adaptive and nonadaptive but optimal item selection also became more prominent further away from the origin (Figure 4).

#### Score Distributions

Contrary to expectations, using the more lenient social desirability balancing criterion didn’t lead to more favorable sample mean scores. The effect sizes of the differences (using strict social desirability balancing as the baseline) were negligible for nonadaptive but optimal conditions (Cohen’s *d* magnitude < 0.10 on all six factors), and actually favored strict social desirability balancing for adaptive conditions (Cohen’s *d* = −0.250 for H, 0.180 for E, −0.178 for X, −0.163 for A, 0.079 for C, and −0.158 for O).

#### Participant Perceptions

Despite clearly stating that the feedback questions were optional and inconsequential, most participants were still motivated enough to answer them (valid *N* = 1,045–1,090 per question). Tables 5 and 6 summarize participants’ responses to questions asking about the approximate frequencies in which they encountered (a) FC pairs of adjectives that were both like them or both unlike them (i.e., similar utility); (b) FC pairs of adjectives that were clearly unmatched in social desirability. For each question, a Kruskal–Wallis rank sum test was conducted to check whether the responses (with “don’t know” responses treated as missing) were significantly different across all four design conditions. Contrary to a priori predictions, participants appeared to share very similar perceptions around item utility (χ^2^ = 2.84, *df* = 3, *p* = .42) as well as social desirability (χ^2^ = 6.70, *df* = 3, *p* = .08).

**Table 5.** The Perceived Frequency of Seeing a Pair of Adjectives With Similar Utility.

Response	Design condition			
	AL	AS	NL	NS
0% of the time	0.4%	1.5%	0.8%	1.6%
25% of the time	29.1%	36.3%	29.8%	28.1%
50% of the time	49.1%	36.7%	40.7%	44.3%
75% of the time	20.0%	22.4%	26.0%	25.3%
100% of the time	1.5%	3.1%	2.7%	0.8%
Do not know	0.0%	0.0%	0.0%	0.0%
*N*	275	259	258	253

*Note.* AL = adaptive with lenient social desirability balancing, AS = adaptive with strict social desirability balancing, NL = nonadaptive but optimal with lenient social desirability balancing, NS = nonadaptive but optimal with strict social desirability balancing.

**Table 6.** The Perceived Frequency of Seeing a Pair of Adjectives With Unmatched Social Desirability.

Response	Design condition			
	AL	AS	NL	NS
0% of the time	1.0%	2.6%	1.5%	0.8%
25% of the time	38.0%	39.9%	35.8%	33.0%
50% of the time	32.4%	32.8%	35.1%	37.5%
75% of the time	19.5%	15.3%	19.2%	21.1%
100% of the time	1.4%	1.9%	3.8%	1.9%
Do not know	7.7%	7.5%	4.5%	5.7%
*N*	287	268	265	261

*Note.* AL = adaptive with lenient social desirability balancing, AS = adaptive with strict social desirability balancing, NL = nonadaptive but optimal with lenient social desirability balancing, NS = nonadaptive but optimal with strict social desirability balancing.

In the last feedback question, participants considered how successful a dishonest candidate might be in inflating scores for the FC instruments (Table 7). Between 3 and 4 out of 10 participants per condition (31.4%–40.3%) expected faking good to be “not at all successful.” Participants’ opinions appeared to be similar across all four design conditions (Kruskal–Wallis rank sum test χ^2^ = 3.14, *df* = 3, *p* = .37).

**Table 7.** Anticipated Success of Intentional Score Inflation in the FC Instruments.

Response	Design condition			
	AL	AS	NL	NS
Not at all successful	31.4%	40.3%	35.4%	36.3%
Somewhat successful	41.8%	40.3%	40.3%	39.3%
Very successful	5.6%	5.1%	8.7%	5.2%
Extremely successful1	1.7%	1.8%	0.8%	0.7%
Don’t know	19.5%	12.5%	14.8%	18.4%
*N*	287	273	263	267

*Note.* AL = adaptive with lenient social desirability balancing, AS = adaptive with strict social desirability balancing, NL = nonadaptive but optimal with lenient social desirability balancing, NS = nonadaptive but optimal with strict social desirability balancing.

### Simulation Study

**Figure 5.**
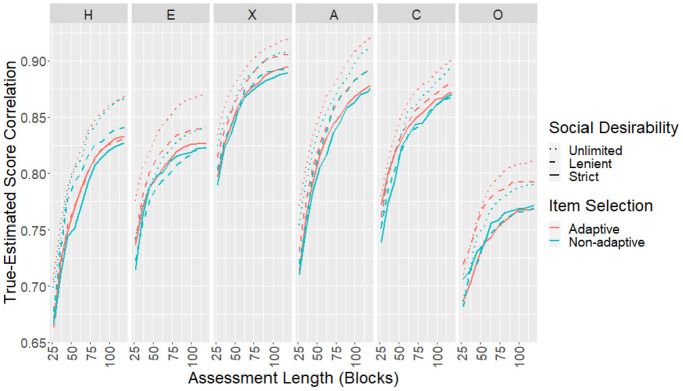
Correlations Between True and Estimated Scores for the Simulated Sample.

**Figure 6.**
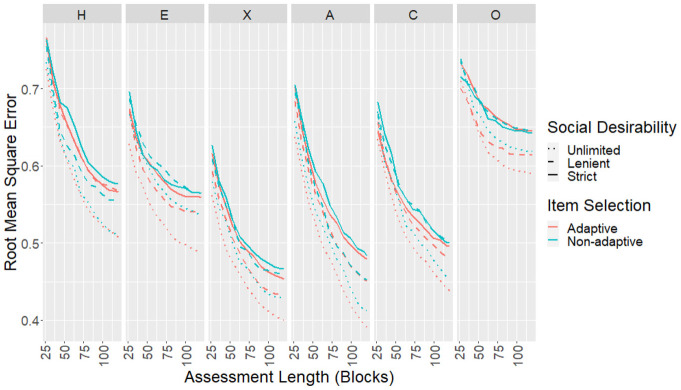
RMSEs Between True and Estimated Scores for the Simulated Sample.

The CORs, RMSEs, and mean SEMs for each scale as the test progressed were plotted. In terms of mean SEMs (Figure 2), the effects of adaptive item selection and social desirability balancing were in line with findings from the empirical study. Results for CORs (Figure 5) and RMSEs (Figure 6) showed similar patterns. Removing the social desirability balancing constraint completely led to slightly better measurement precision in some scales.

## Discussion

This study examined a multidimensional FC CAT using dominance items modeled by the Thurstonian IRT model (Brown & Maydeu-Olivares, 2011), quantifying the empirical implications of adaptive item selection and social desirability balancing criteria on measurement precision, score distributions, and participant perception. To our knowledge, this is the first empirical study of CAT with dominance FC items and normative IRT scoring. The analysis was largely exploratory and the results were mixed.

### Adaptive Item Selection

It was confirmed that adaptive item selection achieved greater measurement precision than nonadaptive but optimal item selection. However, the incremental gain was much smaller than those reported in similar FC CAT literature (e.g., Joo et al., 2019; Stark & Chernyshenko, 2007, 2011; Stark et al., 2012). One contributing factor to this was the choice of baseline reference—while CAT research typically adopted random item selection with some content constraints as the baseline for comparison, this study chose a tougher competitor that incorporated optimal item selection to maximize information gain at the population average. In real-life assessments, random item selection is rarely used, so a nonadaptive but optimal item selection represents a more realistic operational baseline for comparison. In other words, this study explored the practical return on investment when converting an otherwise-optimized static assessment into an adaptive one. Another contributing factor to the small adaptive advantage was the very limited item bank, with each FC assessment using up 240 out of 279 available items, thus greatly limiting the possibility and potential of adaptive item selection toward the end of the assessment sessions. The limiting effect of the item bank was made more severe by its relatively low discrimination parameters (Table 2). As described by Davey and Nering (2002), items with high discriminations are intense “spotlights” that focus on measuring a small region in the trait space, whereas items with low discriminations are less-intense “floodlights” that give less targeted information but over a larger region in the trait space. The bank of HEXACO adjectives had more “floodlights” than “spotlights,” but the latter is needed for CAT to “zoom in” on a candidate’s scores effectively. Therefore, the presence of a large, varied and discriminating item bank would likely be a prerequisite for effective FC CAT.

Although the effects of adaptive item selection were very small (although still consistent and significant) at the sample level, it became more prominent for certain individuals. In particular, score profiles that were further away from the origin/population mean benefited more from adaptive item selection. Furthermore, adaptive item selection was more successful at certain trait values, suggesting that its effectiveness might be highly dependent on the composition of the item pool (e.g., the distribution of item mean utility values and how they overlap between scales, the distribution of item loading and uniqueness parameters and how much information is achievable at each location of the trait space, the proportion of negatively keyed items and how easily they can be slotted into MFC pairs, and how these item bank characteristics interact between scales during test construction), as well as the characteristics of the target candidate population (i.e., how well does the item bank match the candidates’ score distributions). Such interactions made the generalization of results across different item banks particularly difficult, and further studies with different item banks would be desirable to understand FC CAT better.

Interestingly, adaptive item selection did not produce any notable measurement advantages at shorter test lengths compared with an otherwise-optimized static assessment. The lack of improvements at the beginning of assessment despite having plenty of items to choose from was likely due to the unreliability of interim trait estimates. Indeed, despite its bias-reducing qualities, the FC pair format elicits less information per binary response compared with a SS item with a more detailed graded response (Brown & Maydeu-Olivares, 2017). There are multiple implications of this finding in practice. At the simplest level, there might be a test length below which adaptive item selection would not be worthwhile for FC assessments. Instead, it would be more economical to delay adaptive item selection till after a certain test length has been reached (e.g., by administering a fixed optimal test first), and/or make use of other data (e.g., prior information from alternative data sources, initial SS questions) to arrive at more reliable interim trait estimates prior to converting to FC CAT for reducing SEMs for the scales that are still lacking in measurement. Alternatively/in addition, the use of larger FC blocks (e.g., triplets, quads) would result in more information gain per question than pairs (Brown & Maydeu-Olivares, 2017) while also being less demanding on the richness of the item bank (i.e., larger blocks produce more pairwise comparisons per item used), thus allowing faster convergence to reliable interim trait estimates but at the expense of greater computational complexity in item selection and higher cognitive complexity for the candidates. At a more technical level, it will be beneficial to explore item selectors that don’t rely on point estimates, for example, item selectors using the Kullback–Leibler global information concept (Cover & Thomas, 2006; Kullback, 1959; Lehmann & Casella, 1998). The power of item selectors that consider the entire posterior distribution has been demonstrated by past research (e.g., Chang & Ying, 1996; Mulder & van der Linden, 2010; Veldkamp & van der Linden, 2002; C. Wang & Chang, 2010, 2011; Weissman, 2007) and it is reasonable to hypothesize the findings would generalize to FC CAT.

The impact of item selection methodology was largely limited to measurement precision only. Compared with static assessments, adaptive item selection made practically no impact on participant perceptions. Although candidates may hold different views about adaptive and nonadaptive assessments, the actual assessment experience appeared to be largely indistinguishable in practice.

### Social Desirability Balancing

Social desirability balancing is important for ensuring resistance against faking (Krug, 1958). When items are placed into FC pairs, larger desirability differences between them will lead to greater opportunities for socially desirable responding. The threshold at which the “right answer” becomes apparent can be identified through an empirical study that asks participants to purposefully choose the “right answer.” However, a candidate will not necessarily choose the “right answer” even if they can spot it. It is hypothesized that whether a candidate will choose the “right answer” over the real answer depends on their character, the size of the difference in social desirability of items, and the stakes of the assessment (e.g., Birkeland et al., 2006). It follows that the threshold at which socially desirable responding becomes a problem could vary depending on the assessment setting and purpose, with high-stakes assessments demanding stricter social desirability balancing, while low-stakes assessments being able to use more lenient criteria. For a low- to medium-stakes assessment setting as in the current study (i.e., assessment results were inconsequential for the participants, but most of them were likely answering the questions as if they were applying for a job so as to practice for their actual pre-employment assessments), the lenient criteria used appeared adequate at the sample level (i.e., it did not lead to more favorable sample mean scores than the strict condition), and might possibly be relaxed even further without impairing fake resistance of the FC measures. However, at the individual level, some candidates might still be able to inflate their scores. In practice, care should be taken to check the prevalence of faking success at the individual level when deciding whether a social desirability balancing criterion is strict enough.

Note that setting the social desirability balancing threshold is a balancing act—there is a trade-off between the strictness of social desirability balancing and the effectiveness of adaptive item selection. A more stringent social desirability balancing criterion inevitably reduces the number of acceptable FC blocks, therefore limiting the freedom of adaptive item selection. In this study, the strict social desirability balancing criterion indeed led to slightly worse measurement precision. This trade-off is especially relevant for high-stakes assessments, where stricter social desirability balancing is needed for better fake resistance. If the item bank is not large and varied enough, the strict social desirability balancing requirement may negate any measurement improvement potential of adaptive item selection. In such a situation, the benefits of adaptive item selection are mainly around enhancing test security (i.e., by creating different question sequences for different candidates).

In lieu of actual item social desirability estimates, this study adopted the item mean as a proxy. This is frequently done in faking research (e.g., Jackson et al., 2000; Watrin et al., 2019) and it has been shown in a meta-analysis that balancing on the item mean (“extremity”) significantly reduces the faking effects (Cao & Drasgow, 2019). Although it is customary to use the item mean utility as a proxy for social desirability, it can lead to some undesirable effects especially in CAT. Placing the social desirability constraint T on the item mean differences means that a FC pair {i,k} is only allowed if $-T\leq \mu_{i}-\mu_{k}\leq T$ . Meanwhile, the information from the pair {i,k} peaks when $\mu_{i}-\mu_{k}+{\lambda_{i}}_{s_{i}}\eta_{s_{i}}-{\lambda_{k}}_{s_{k}}\eta_{s_{k}}=0$ (see Online Supplement), which defines the score η combinations that this pair is most effective at measuring. Therefore, FC pairs satisfying the social desirability balancing criterion are best at measuring η in the region $-T\leq -{\lambda_{i}}_{s_{i}}\eta_{s_{i}}+{\lambda_{k}}_{s_{k}}\eta_{s_{k}}\leq T$ , which can be represented graphically in the 2-dimensional space for traits $\eta_{s_{i}}$ and $\eta_{s_{k}}$ as a band of width $T/|{\lambda_{k}}_{s_{k}}|$ around the line $\eta_{s_{k}}=({\lambda_{i}}_{s_{i}}/{\lambda_{k}}_{s_{k}})\eta_{s_{i}}$ . Different pairs give different values for ${\lambda_{i}}_{s_{i}}/{\lambda_{k}}_{s_{k}}$ and $T/|{\lambda_{k}}_{s_{k}}|$ , leading to bands of different slopes and widths that all intersect at the origin. For small item banks, these bands may not fully cover all important regions in this two-dimensional space, especially if the value T is strict/ small (which makes the bands narrower). Even for large item banks, there is a practical limit to the values of ${\lambda_{i}}_{s_{i}}/{\lambda_{k}}_{s_{k}}$ , and thus certain regions in the two-dimensional space may still be uncovered. The score regions not covered by these bands can still be measured, but less effectively because there are no FC pairs that satisfy the social desirability constraint and target those traits and regions specifically. This limiting effect on item selection may be alleviated to some degree if many traits are being measured, giving multiple pairs of traits to select from, each with its own covered regions in the corresponding two-dimensional space. The limiting effect may be further alleviated if actual social desirability ratings are used, thereby breaking the link between item parameters and social desirability balancing. Although, given the typically high correlations between social desirability and item means (Kuncel & Tellegen, 2009), the increase in item selection freedom may still be somewhat limited. Further research should explore the use of actual social desirability estimates and how they interact with adaptive item selection, and whether any new dynamics arises compared with when using item mean as a proxy.

Social desirability balancing criteria had no notable impact on participant perceptions, suggesting that the assessment experience was comparable across design conditions.

## Limitations

On the micro level, a number of limitations have been highlighted and discussed throughout the article. On the macro level, this empirical study explored only one specific instance of multidimensional FC assessment using dominance items: It made use of a specific HEXACO item bank; it explored the effect of only one content rule (i.e., social desirability balancing criteria); it adopted the simplest pair format which is not the most information-efficient FC design; and it adopted an item selector that relies heavily on interim point estimates of trait values. Also, the instruments were completed under only one specific assessment setting (i.e., practice for pre-employment assessments). Given the numerous design possibilities and assessment situations, it would be unwise to conclude the merits of FC CAT with dominance items based on the findings of this one study. To further the understanding of FC CAT with dominance items, it would be necessary to conduct more empirical studies with varying scale constructs, item banks, IRT models, assessment designs, respondent populations, and so on. Nevertheless, this study provided an initial exploratory baseline for furthering empirical research on FC CAT with dominance items.

## Implications

The development of a good FC CAT is a journey that requires considerations from many angles. As an analogy, for a vehicle to reach its destination, it requires a powerful engine (the FC CAT algorithm), sufficient amount of fuel (the item bank), adequate driver steering controls (the computerized assessment delivery platform), and a map of the terrain (the psychological constructs being measured). Through close empirical examination of a “prototype vehicle,” this study highlighted a number of important psychometric and practical considerations and furthered our understandings of FC assessments and CAT.

First, this study extended the literature on FC assessments using dominance items and the TIRT model (e.g., the Motivational Value Systems Questionnaire by Merk et al. (2017)), informing research and practice for the design and deployment of such assessments regardless of whether they are adaptive or not. Second, findings of this study also inform FC assessment development even if the TIRT model isn’t adopted (e.g., see meta-analysis of FC measures by Salgado and colleagues (2014, 2015, 2017)), providing empirical insight into respondent behaviors and reactions with respect to the FC response format in general. Finally, as many items were developed under the dominance rather than ideal-point paradigm (e.g., the International Personality Item Pool; Goldberg et al., 2006), improving the understanding of FC CAT methodologies for dominance items opens up more opportunities for leveraging such legacy items for future FC CAT applications.

## Supplemental Material

sj-docx-1-epm-10.1177_00131644221077637 – Supplemental material for Multidimensional Forced-Choice CAT With Dominance Items: An Empirical Comparison With Optimal Static Testing Under Different Desirability Matching Click here for additional data file. Supplemental material, sj-docx-1-epm-10.1177_00131644221077637 for Multidimensional Forced-Choice CAT With Dominance Items: An Empirical Comparison With Optimal Static Testing Under Different Desirability Matching by Yin Lin, Anna Brown and Paul Williams in Educational and Psychological Measurement

## Appendix A

### Technical Details on FC Test Assembly

Different approaches can be taken when setting up a FC CAT (Table A1). In a “fixed blocks” setup, all available FC blocks are predetermined. In this setup, the FC response format does not introduce additional psychometric complexities around item selection, so the CAT functions in the same way as other multidimensional CATs with a predetermined item bank, which is a relatively well-researched area in the literature. This study, however, adopted a more flexible “dynamic blocks” setup, where FC blocks are created on the fly.

**Table A1.** Procedures for Creating a FC CAT.

Approach	Procedure
Fixed blocks	1. A pool of FC blocks are constructed to satisfy content rules (e.g., social desirability balancing). 2. For calibration, the FC blocks are administered as-is, and IRT parameters are estimated against blocks. 3. Test assembly picks from the pool of pre-existing FC blocks. No new blocks can be made from existing blocks.
Dynamic blocks	1. A pool of items are written to satisfy content rules (e.g., no double negative). 2. For calibration, the items are administered in a SS format, and IRT parameters are estimated against items. 3. There are no pre-existing FC blocks. Test assembly needs to create new FC blocks by picking from the item pool, subject to requirements (e.g., whether to make blocks of 2 or 3 items) and constraints (e.g., social desirability balancing). Properties of a FC block are deduced from properties of its constituting items.

*Note.* FC = forced-choice; IRT = item response theory; SS = single-stimulus.

The “dynamic blocks” setup is more efficient than the “fixed blocks” setup. For example, suppose we have 2 scales with 10 items each, giving 10 × 10 = 100 multidimensional FC pairs. The “fixed blocks” setup requires the parameters for all 100 FC blocks to be calibrated, whereas the “dynamic blocks” setup only requires the parameters for the 20 source items to be estimated. Although the psychological interpretation of an item may fluctuate depending on what other item it is paired with, such small fluctuations have very limited effect on the final observed scores, supporting the assumption of invariant item parameters across FC blocks (Lin & Brown, 2017; Morillo et al., 2019). The item parameter invariance assumption allowed the properties of a FC block to be deduced from the properties of its constituting items, enabling the more efficient “dynamic blocks” setup. The technical details of how to calibrate items from SS data to be used in FC formats are explained in more detail in the “Method” section.

Once the item bank and associated parameters are established, test assembly selects on block at a time:

1. Determine the list of remaining/unadministered items that can still be selected to create the next FC block.
2. Create all possible FC blocks from the remaining items.
3. Remove the FC blocks not meeting content requirements (e.g., unidimensional vs. multidimensional, social desirability balancing).
4. Model the remaining FC blocks using properties of their constituting items (i.e., Equation 1), compute information measures (e.g., A-optimality) based on interim trait estimates (starting from the origin).
5. Ranking the FC blocks according to the information measures and select the best one to add to the test.
6. Administer the selected FC block and collect responses.
7. Update interim trait estimates (or skip this step for the nonadaptive but optimal conditions).
8. Repeat until the desired test length is achieved.

Note that it is important to assemble FC test “block-wise” (i.e., choosing multiple items to form a block at each step) rather than choosing one item at a time. This is because the way items are assembled into blocks have an impact on the resulting information gain. Even if the exact same items are used across two FC forms, the resulting measurement precisions can be different if the items are assembled into FC blocks differently.
