# Supplementary material for: Multidimensional Forced-Choice CAT With Dominance Items: An Empirical Comparison With Optimal Static Testing Under Different Desirability Matching
Source: Educ Psychol Meas. 2022 Mar 7;83(2):322–50. doi: 10.1177/00131644221077637 (PMC9972128; doi:10.1177/00131644221077637)
Supplement: sj-docx-1-epm-10.1177_00131644221077637 – Supplemental material for Multidimensional Forced-Choice CAT With Dominance Items: An Empirical Comparison With Optimal Static Testing Under Different Desirability Matching [file sj-docx-1-epm-10.1177_00131644221077637.docx]

# SUPPLEMENT

# Appendix B. Information Gain and Item Mean Utility Differences

This study adopted the item mean utility parameter as a proxy for item social desirability. Figure B1 plots the information gain from pairs with item mean utility differences of 0, 1 and 2 respectively, while fixing all other item parameters. It can be seen that, while the item mean utility parameters had no effect on the maximum amount of information gain, they had an impact on the location of where that maximum occurs.

| Parameters | Information for Scale 1 | Information for Scale 2 |
| --- | --- | --- |
| $\mu_{1}=0$  $\mu_{2}=0$ | 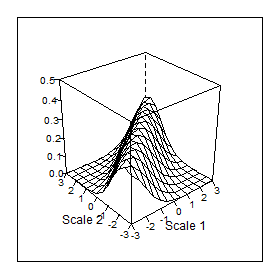 | 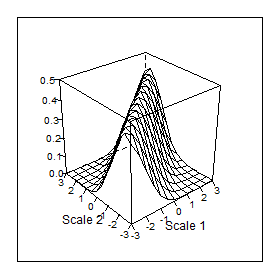 |
| $\mu_{1}=-0.5$  $\mu_{2}=0.5$ | 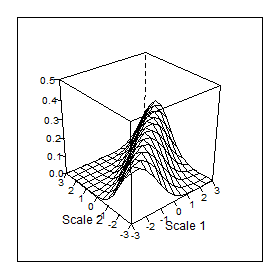 | 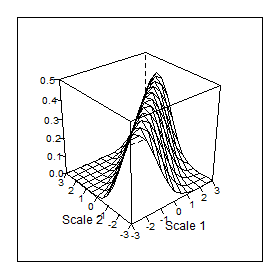 |
| $\mu_{1}=-1$  $\mu_{2}=1$ | 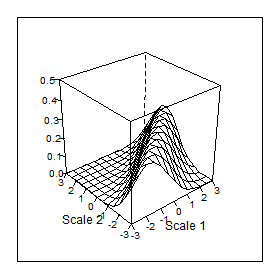 | 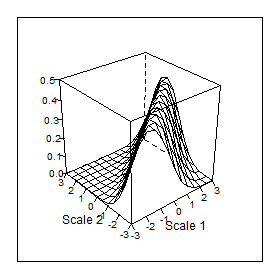 |

Figure B1. Information gain from a pair with varying item mean utility differences and ${\lambda_{1}}_{s_{1}}=1.2$, ${\lambda_{2}}_{s_{2}}=1.4$, $\psi_{1}^{2}=\psi_{2}^{2}=1$, and a scale intercorrelation of 0.2.

# Appendix C. Information Plots for Example Pairs

Figures C1 to C4 provide the information plots for the first and last FC pairs from the non-adaptive instruments. The parameters of the constituting items are summarized in Table C1. As a consequence of the optimal form design (i.e., always picking the next FC pair to maximize information gain at the origin), the last parts provided less information in general compared to the first pairs.

Table C1. Example FC pairs from the non-adaptive instruments

| **Social desirability balancing** | **Pair Number** | **Item** | $s_{i}$ | $\mu_{i}$ | ${\lambda_{i}}_{s_{i}}$ | $\psi_{i}^{2}$ | ${{\lambda_{i}}_{s_{i}}}/{\psi_{i}}$ |
| --- | --- | --- | --- | --- | --- | --- | --- |
| Strict | 1 | A247 | X | 3.586 | −0.733 | 1.544 | −0.590 |
|  |  | A20 | O | 4.026 | 0.889 | 1.275 | 0.787 |
| Lenient | 1 | A236 | X | 3.233 | −0.810 | 1.230 | −0.730 |
|  |  | A20 | O | 4.026 | 0.889 | 1.275 | 0.787 |
| Strict | 120 | A64 | A | 5.376 | 0.436 | 0.526 | 0.601 |
|  |  | A222 | C | 5.159 | 0.360 | 0.746 | 0.417 |
| Lenient | 120 | A235 | A | 2.344 | −0.650 | 1.461 | −0.538 |
|  |  | A117 | C | 1.996 | −0.482 | 1.182 | −0.443 |

| 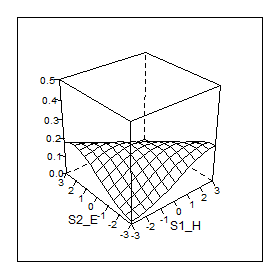 | 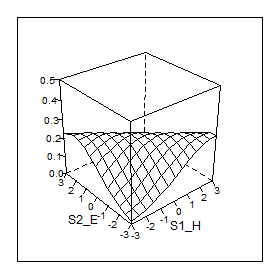 |
| --- | --- |

Figure C1. Information for scales X (left) and O (right) from pair {A247, A20}

| 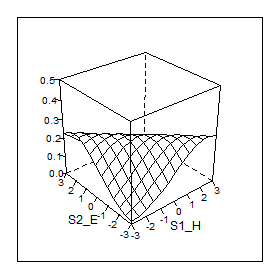 | 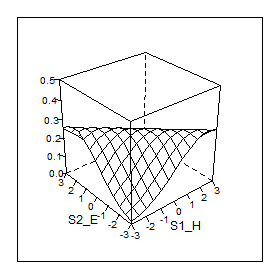 |
| --- | --- |

Figure C2. Information for scales X (left) and O (right) from pair {A236, A20}

| 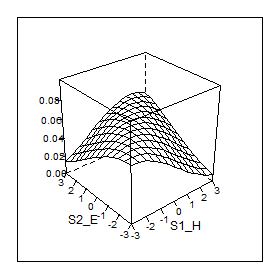 | 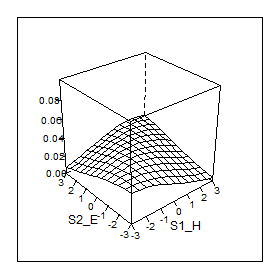 |
| --- | --- |

Figure C3. Information for scales A (left) and C (right) from pair {A64, A222}

| 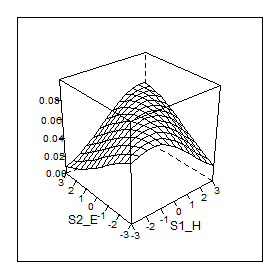 | 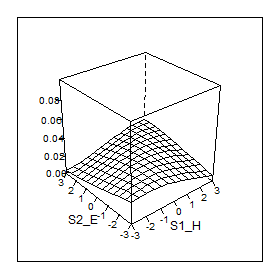 |
| --- | --- |

Figure C4. Information for scales A (left) and C (right) from pair {A235, A117}
